# No association between polymorphisms in the *BDNF *gene and age at onset in Huntington disease

**DOI:** 10.1186/1471-2350-7-79

**Published:** 2006-11-10

**Authors:** Maren Mai, Amer D Akkad, Stefan Wieczorek, Carsten Saft, Jürgen Andrich, Peter H Kraus, Jörg T Epplen, Larissa Arning

**Affiliations:** 1 Department of Human Genetics, Ruhr-University, 44780 Bochum, Germany; 2 Department of Neurology, St. Josef-Hospital, Ruhr-University, 44791 Bochum, Germany

## Abstract

**Background:**

Recent evidence suggests that brain-derived neurotrophic factor (BDNF) is an attractive candidate for modifying age at onset (AO) in Huntington disease (HD). In particular, the functional Val66Met polymorphism appeared to exert a significant effect. Here we evaluate *BDNF *variability with respect to AO of HD using markers that represent the entire locus.

**Methods:**

Five selected tagging polymorphisms were genotyped across a 65 kb region comprising the *BDNF *gene in a well established cohort of 250 unrelated German HD patients.

**Results:**

Addition of *BDNF *genotype variations or one of the marker haplotypes to the effect of CAG repeat lengths did not affect the variance of the AO.

**Conclusion:**

We were unable to verify a recently reported association between the functional Val66Met polymorphism in the *BDNF *gene and AO in HD. From our findings, we conclude that neither sequence variations in nor near the gene contribute significantly to the variance of AO.

## Background

Conclusive evidence indicates that brain-derived neurotrophic factor (BDNF) plays a pivotal role in the pathophysiology of Huntington disease (HD). As the protein huntingtin (htt) directly modulates the expression of neuron-restrictive silencer factor (NRSF)-controlled genes, wild type (wt) htt stimulates the production of BDNF, whereas mutant htt causes the opposite effect [1]. It has been shown recently in transgenic mice that BDNF has an impact on the age at onset (AO) and the severity of motor dysfunction by controlling survival of striatal projection neurons [2].

The *BDNF *gene consists of five alternatively spliced 5' exons and one major 3' exon producing at least six *BDNF *transcripts leading to three pre-proprotein isoforms which differ in the lengths of the signal peptides. Sequence variations in *BDNF *may therefore lead to variations in gene expression or protein metabolism causing selective neuronal vulnerability. The single nucleotide polymorphism (SNP) rs6265, producing a valine-to-methionine substitution at codon 66 (Val66Met) in the human *BDNF *gene appears to exert an effect on intracellular trafficking and activity-dependent secretion of BDNF [3]. Furthermore, Met-BDNF carriers demonstrate substantial relative decreases in hippocampal volume, and Val/Met-*BDNF *affects the volume of gray matter in the cerebral neocortex of normal humans. Finally Met-BDNF is associated with volume reductions primarily in the lateral convexity of the prefrontal cortex [4]. Thus, the Val66Met polymorphism may be a modifying genetic factor in the expression of a number of normal and abnormal brain conditions, and therefore represents a good candidate gene for modifying AO in HD. In this context, several associations between *BDNF *polymorphisms and neurological and psychiatric disorders have been reported [5]. In addition, a recent study demonstrated that HD patients heterozygous for the Val66Met polymorphism present a later AO than homozygous carriers of Val-BDNF [6].

In this study, we investigated the relation between the *BDNF *gene and the AO of HD using genetic markers that represent the overall variability at this locus.

## Methods

We selected tagging polymorphisms from the *BDNF *gene (rs6265, rs11030104, rs7103873, rs2049046 and rs12273363) based on HapMap data. We examined the associations between *BDNF *polymorphisms and motor AO of HD in 250 unrelated patients with clinical diagnosis of HD as recruited from the Huntington Center (HZ) NRW, Bochum (Germany) [7]. Informed consent was obtained and the institutional Ethics Committee of Bochum Ruhr-University approved this study.

The expanded CAG repeats explained 50.9% of the variance in AO in this cohort. The potential influence of certain genotypes on AO was calculated by linear regression, in which R^2 ^illustrates the relative improvement of the regression model when the various genotypes are considered in addition to the expanded CAG block in the *huntingtin *gene.

## Results and Discussion

**Figure 1:**
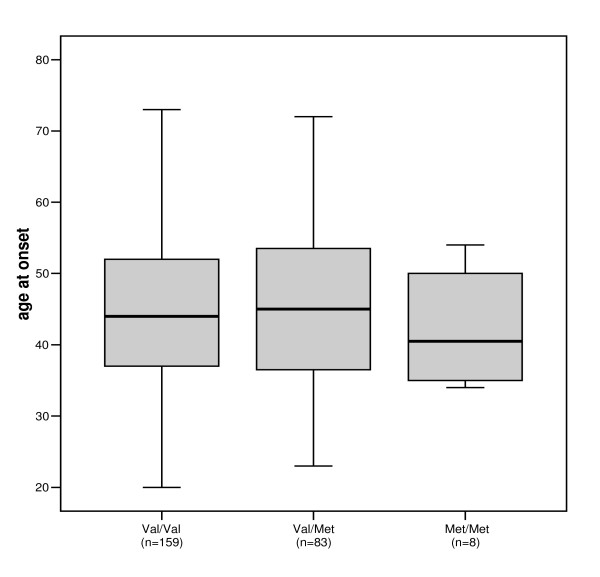
Relationship between *BDNF *Val66Met (rs6265) genotypes and age at onset (AO) for 250 Huntington disease patients. For each genotype, the median AO is represented as a black bar, the quartile is shown as a solid box, and the range is indicated by the margins.

LD analysis revealed strong LD between all neighboring markers. Patients carrying different genotypes and haplotypes, respectively, showed no differences in AO as evidenced in the box plot of Val66Met genotypes and AO (figure 1). Addition of *BDNF *genotype variations or one of the five marker haplotypes to the effect of CAG repeat lengths resulted in no significant increase of the R^2 ^value. The same effect was evident in a sub-group of patients (n = 194) with higher variance in AO (CAG repeat range 40–45; R^2 ^= 0.36). Observed frequencies for all five SNPs (table 1) were in Hardy-Weinberg equilibrium. Our results clearly indicate that genetic variations in the *BDNF *gene do not influence the variance of AO in HD in German patients in contrast to the results of Alberch and collaborators [6]. Several reasons may explain the discrepancy between these results. The previously reported association could have resulted from a type I error. Possible selection bias due to admixture may have been due to inclusion of DNA samples of relatives, the exclusion of which has not fully been detailed in the paper. Further evidence for such an assumption is implied by the very low R^2 ^(0.11) in the interval between 42 and 49 CAG repeats. Alternatively, true association could exist only in groups of a specific ethnic origin and, presumably, a similar genetic background. While this manuscript was under review two other independent reports have been published by two different groups leading to the same conclusion indicating no association between the Val66Met genotype and variation in AO [8,9], nor three other *BDNF *SNPs [9].

**Table 1:** Allele and genotype frequencies of the *BDNF *polymorphisms

	Allele counts (Frequency-%)		Genotype counts (Frequency-%)		
rs6265 (Val66Met)	Val 401 (80.0)	Met 99 (20.0)	Val/Val 159 (64.0)	Val/Met 83 (33.0)	Met/Met 8 (3.0)
rs11030104	A 396 (79.0)	G 104 (21.0)	AA 155 (62.0)	AG 86 (34.0)	GG 9 (4.0)
rs7103873	C 257 (51.0)	G 243 (49.0)	CC 70 (28.0)	CG 117(47.0)	GG 63 (25.0)
rs2049046	T 261 (52.0)	A 239 (48.0)	AA 75 (30.0)	AT 111(44.0)	TT 64 (26.0)
rs12273363	T 421 (84.0)	C 79 (16.0)	TT 173 (69.0)	TC 75 (30.0)	CC 2 (1.0)

## Conclusion

In our association study between common polymorphisms that represent the entire variability of the *BDNF *gene and the variance in motor AO in HD, we were unable to verify the reported association between a single polymorphism at the *BDNF *gene and AO in HD.

## Competing interests

The author(s) declare that they have no competing interests.

## Authors' contributions

MM carried out the molecular genetic studies. ADA established the assays. SW helped writing the manuscript. JA and CS had ascertained the clinical status of the patients. PHK participated in the data analysis. LA initiated the study and drafted the manuscript. JTE participated in the study design, the coordination and finalized the analyses as well as the paper. All authors read and approved the final manuscript.

## Pre-publication history

The pre-publication history for this paper can be accessed here:



## References

[B1] Zuccato C, Tartari M, Crotti A, Goffredo D, Valenza M, Conti L, Cataudella T, Leavitt BR, Hayden MR, Timmusk T, Rigamonti D, Cattaneo E (2003). Huntingtin interacts with REST/NRSF to modulate the transcription of NRSE-controlled neuronal genes. Nat Genet.

[B2] Canals JM, Pineda JR, Torres-Peraza JF, Bosch M, Martin-Ibanez R, Munoz MT, Mengod G, Ernfors P, Alberch J (2004). Brain-derived neurotrophic factor regulates the onset and severity of motor dysfunction associated with enkephalinergic neuronal degeneration in Huntington's disease. J Neurosci.

[B3] Egan MF, Kojima M, Callicott JH, Goldberg TE, Kolachana BS, Bertolino A, Zaitsev E, Gold B, Goldman D, Dean M, Lu B, Weinberger DR (2003). The BDNF val66met polymorphism affects activity-dependent secretion of BDNF and human memory and hippocampal function. Cell.

[B4] Pezawas L, Verchinski BA, Mattay VS, Callicott JH, Kolachana BS, Straub RE, Egan MF, Meyer-Lindenberg A, Weinberger DR (2004). The brain-derived neurotrophic factor val66met polymorphism and variation in human cortical morphology. J Neurosci.

[B5] Zhang H, Ozbay F, Lappalainen J, Kranzler HR, van Dyck CH, Charney DS, Price LH, Southwick S, Yang BZ, Rasmussen A, Gelernter J (2006). Brain derived neurotrophic factor (BDNF) gene variants and Alzheimer's disease, affective disorders, posttraumatic stress disorder, schizophrenia, and substance dependence. Am J Med Genet (Neuropsychiatr Genet).

[B6] Alberch J, Lopez M, Badenas C, Carrasco JL, Mila M, Munoz E, Canals JM (2005). Association between BDNF Val66Met polymorphism and age at onset in Huntington disease. Neurology.

[B7] Arning L, Kraus PH, Valentin S, Saft C, Epplen JT (2005). *NR2A *and *NR2B *receptor gene variations modify age at onset in Huntington disease. Neurogenet.

[B8] Di Maria E, Marasco A, Tartari M, Ciotti P, Abbruzzese G, Novelli G, Bellone E, Cattaneo E, Mandich P (2006). No evidence of association between BDNF gene variants and age-at-onset of Huntington's disease. Neurobiol Dis.

[B9] Kishikawa S, Li JL, Gillis T, Hakky MM, Warby S, Hayden M, Macdonald ME, Myers RH, Gusella JF (2006). Brain-derived neurotrophic factor does not influence age at neurologic onset of Huntington's disease. Neurobiol Dis.

